# Phage Therapy Beyond Static Pharmaceuticals: A Framework for Controlled Evolutionary Platforms

**DOI:** 10.3390/v18050534

**Published:** 2026-05-01

**Authors:** Hidetomo Iwano, Jumpei Fujiki, Tomohiro Nakamura

**Affiliations:** Laboratory of Veterinary Biochemistry, School of Veterinary Medicine, Rakuno Gakuen University, Ebetsu 069-8501, Japan; j-fujiki@rakuno.ac.jp (J.F.); t-nakamura@azabu-u.ac.jp (T.N.)

**Keywords:** bacteriophage, phage therapy, antimicrobial resistance, evolutionary dynamics, population heterogeneity, controlled evolutionary platforms, regulatory science

## Abstract

Rising antimicrobial resistance has revived global interest in phage therapy, yet its transition to standard clinical practice remains slow. This challenge is not solely due to a lack of efficacy. Instead, we face a fundamental conceptual barrier caused by an “evaluation mismatch.” Traditional regulations treat phages as static chemical molecules—like taking a “snapshot.” However, biologically, phages are dynamic, evolving populations—more like a living “movie.” In this review, we use Schrödinger’s cat metaphor to explain this reality: phage variability is not a defect, but an essential feature. To bridge this gap, we propose a Controlled Evolutionary Platform. By distinguishing between a fixed “Safety Core” and a fluctuating “Adaptive Periphery,” we can manage viral evolution rather than trying to stop it. Ultimately, to integrate phages into modern medicine, we must redefine “consistency”: shifting our focus from preserving a fixed genetic sequence to ensuring the reliable performance of population dynamics.

## 1. Introduction

### 1.1. The Historical Trajectory: From Discovery to Renaissance

The story of phage therapy began more than a century ago, marked by a dramatic pendulum swing from discovery to abandonment, and finally to resurgence. Bacteriophages were independently discovered by Frederick Twort in 1915 [1] and Félix d’Herelle in 1917 [2]. Initially, the scientific community welcomed these “bacteria eaters” with enthusiasm, viewing them as a potential cure for bacterial infections in an era with few other options [3,4,5,6]. However, the path of medical history bifurcated in the mid-20th century. With the mass production of penicillin, the world entered the “Golden Age of Antibiotics.” The allure of these drugs extended beyond human medicine when their ability to promote growth in livestock was discovered in the late 1940s [7,8]. This pivotal finding led to the widespread, sub-therapeutic use of antibiotics in agriculture, driving global consumption to tens of thousands of tons annually [9].

Concerns about the consequences of this massive usage arose relatively early. In 1969, the United Kingdom issued the “Swann Report,” which recommended restricting the use of antibiotics for growth promotion due to the risk of cross-resistance [10]. This laid the groundwork for future regulations, culminating in the European Union’s total ban on antibiotic growth promoters in 2006 [11]. Despite these regulatory milestones in the West, global consumption continues to skyrocket, driven largely by rising demand for animal protein in low- and middle-income countries [12,13,14]. Today, we face the repercussions of this history. We have entered a “post-antibiotic era” characterized by a “dry pipeline” of new drugs and the relentless spread of multidrug-resistant (MDR) pathogens [15,16,17]. This crisis is not limited to hospitals; resistance genes circulate dynamically across humans, animals, and the environment, necessitating a unified “One Health” approach [18,19]. The economic stakes are staggering: without effective intervention, AMR could result in 10 million deaths annually and cost the global economy up to 100 trillion USD by 2050 [20,21]. Recent large-scale global analyses have further quantified the burden of antimicrobial resistance, demonstrating that resistant infections are associated with substantial mortality and represent a major global health challenge [22]. These alarming findings, together with forecasts of future AMR burden [23], have forced the global medical community to revisit the discarded alternative: the bacteriophage [24,25].

### 1.2. Phage Therapy at a Conceptual Crossroads

Despite this urgent need and a century-long history of use, modern clinical translation of phage therapy has been slow and uneven [26,27]. While high-profile compassionate use cases have demonstrated life-saving potential in otherwise untreatable infections [28,29], large-scale randomized clinical trials—such as the PhagoBurn study—have reported limited efficacy, often due to factors including low phage titers, formulation instability, and mismatches between phage preparations and target bacterial populations [30,31]. Importantly, a growing body of recent clinical and experimental studies has demonstrated the safety and potential efficacy of phage therapy across diverse contexts, including in vivo evolution studies and biofilm-associated systems (e.g., Castledine et al., 2022 [32]; Simmons et al., 2018 [33]), as well as compassionate use programs and emerging clinical treatment frameworks (e.g., Dedrick et al., 2023 [34]; Khatami et al., 2023 [35]). Collectively, these studies consistently indicate that the current limitations of phage therapy do not arise from a lack of biological activity per se, but rather from challenges in aligning therapeutic design, evaluation, and regulatory frameworks. A fundamental bottleneck underlying these challenges is that phages continue to be evaluated within conceptual and regulatory frameworks originally developed for static, chemically defined drugs [36,37,38]. This “static drug model” closely mirrors the paradigm established for antibiotics, in which active substances are chemically defined, stable, and expected to remain invariant across manufacturing batches and clinical use. While many experimental studies actively exploit phage diversity and coevolutionary dynamics to improve therapeutic outcomes, such approaches remain only partially reflected in current regulatory and evaluation frameworks, which continue to emphasize fixed composition and reproducibility. In contrast, phages are self-replicating and evolving biological entities whose therapeutic effects emerge from interactions among heterogeneous populations, bacterial hosts, and ecological context [39,40,41]. As a result, discrepancies frequently arise between experimental measurements, clinical outcomes, and regulatory expectations, highlighting a broader mismatch between dynamic biological systems and static evaluation frameworks [42]. From a regulatory perspective, a central challenge lies in the classification of phage therapy. Unlike conventional therapeutics, phages do not fit neatly into existing categories such as small-molecule drugs or classical biologics. Instead, they occupy an intermediate position between biological medicinal products, adaptive biological systems, and ecological interventions. Furthermore, regulatory expectations differ significantly across application domains. Human therapeutic use requires stringent clinical validation and pharmacovigilance, whereas veterinary and agricultural applications may prioritize population-level efficacy and environmental safety. Environmental applications introduce additional considerations related to ecological impact and horizontal gene transfer. These differences highlight the need for a flexible and context-dependent regulatory framework.

**Figure 1:**
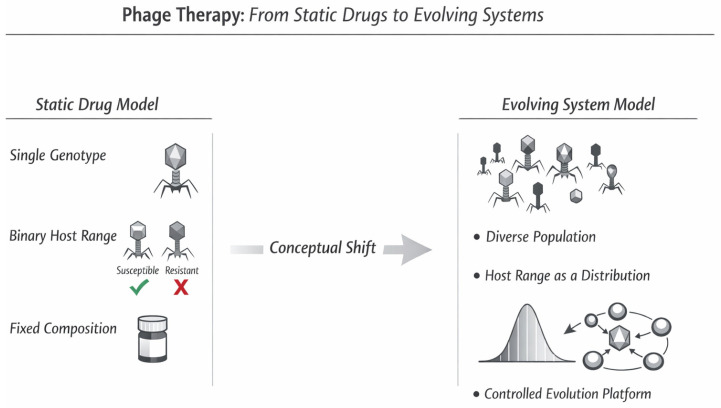
Phage therapy: a conceptual shift from static drug models to evolving system models.

Taken together, these considerations suggest that the current bottleneck in phage therapy is not solely technological, but conceptual. The mismatch between static evaluation frameworks and dynamic biological systems creates a fundamental barrier to clinical translation. Therefore, the purpose of this review is not simply to summarize recent advances in phage therapy, but to integrate emerging evidence and reframe its conceptual foundation (Figure 1). In this context, phages may be more appropriately understood not as uniform medicinal products, but as population-based therapeutic systems whose efficacy, stability, and resistance dynamics are inherently probabilistic. By integrating insights from plaque biology, evolutionary ecology, formulation science, and regulatory analysis, this review proposes a conceptual framework in which phage therapy can be reinterpreted as a controlled evolutionary system, rather than a static pharmaceutical product.

Conventional frameworks have historically treated bacteriophages as static, homogeneous medicinal products characterized by fixed identity, binary host range, and stable composition (left). In contrast, this review proposes reframing phage therapy as a population-based system in which therapeutic properties emerge from heterogeneous phage populations interacting with bacterial hosts and their environments (right). In this framework, host range is interpreted as a probability distribution rather than a binary attribute, and therapeutic updating is achieved through controlled evolutionary platforms that allow adaptive change while maintaining safety, potency, and predictability. This schematic illustrates the proposed conceptual redefinition of phage identity and efficacy, rather than a quantitative or mechanistic comparison.

## 2. Rethinking the Plaque: From Clonal Units to Structured Populations

### 2.1. The Plaque as a Microcosm

**Figure 2:**
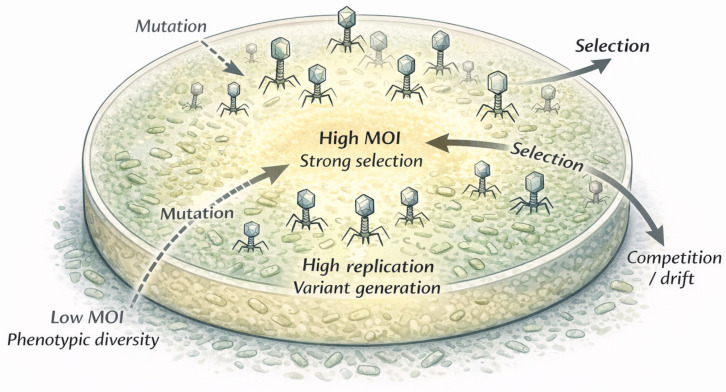
Plaque formation as a dynamic, spatially structured phage population. While conventionally viewed as products of uniform clonal expansion from a single phage particle, plaques instead comprise heterogeneous phage populations structured by spatial gradients in multiplicity of infection, nutrient availability, and host physiological state. Distinct zones within a plaque experience different selective regimes, driving the continuous generation, competition, and selection of genetic and phenotypic variants. Regions of high MOI are associated with strong selection, whereas peripheral regions of low MOI permit higher phenotypic diversity through mutation and drift. Consequently, phenotypic properties such as host range and killing efficacy should be interpreted as emergent population-level distributions rather than fixed attributes of individual genotypes. This schematic illustrates qualitative biological dynamics underlying plaque formation and is not intended as a quantitative or kinetic model.

To understand why this shift is necessary, we must examine the most fundamental unit of phage biology: the plaque. This section does not introduce new experimental data, but rather synthesizes existing findings from multiple studies to provide a conceptual framework for understanding plaque dynamics. Conventionally, a plaque is viewed as a uniform zone of lysis derived from the clonal expansion of a single viral particle [43,44]. In this traditional view, the plaque serves primarily as a quantitative tool—a simple readout of viral titer. However, this interpretation may oversimplify the complex biological processes occurring within plaques. As illustrated in Figure 2, a growing body of experimental and theoretical studies suggests that plaques can be interpreted as spatially structured and dynamically evolving systems.

Within this confined space, phage populations interact with bacterial hosts under gradients of resource availability, infection multiplicity, and physiological state. These interactions give rise to heterogeneous population dynamics that are consistent with previous observations of diffusion-limited propagation and spatially structured infection dynamics.

### 2.2. Anatomy of a Plaque: Gradients and Physical Constraints

The structured organization of plaques arises from spatial gradients and physical constraints that govern phage movement and replication. As schematically shown in Figure 2, a developing plaque contains distinct regions that impose different selective pressures on the phage population [40].

- The Center (Resource-Depleted Zone): The center represents a region of high phage density where host cells are largely depleted, dead, or metabolically inactive, thereby limiting further replication.
- The Periphery (Active Front): At the expanding edge, phages encounter a low multiplicity of infection (MOI) and interact with metabolically active, growing host cells [33,45].

Table 1 summarizes the ecological and evolutionary contrasts between the center and periphery of a phage plaque. The plaque center is characterized by high phage density and host depletion, whereas the periphery represents an active infection front with lower multiplicity of infection (MOI) and higher host availability. These spatial differences create distinct selective environments that shape phage population dynamics and phenotypic heterogeneity.

Crucially, the movement of phages within this structured environment is governed by physical constraints distinct from those in liquid culture. Hu, Miyanaga, and Tanji have quantitatively demonstrated that the effective diffusion coefficient of phages is significantly attenuated within gel matrices and biofilm models compared to free solution [47,48]. This restricted diffusion creates local isolation, preventing rapid population mixing and reinforcing the spatial gradients described above [49,50].

### 2.3. Emergent Properties and Evolution

These spatial gradients—sustained by the physical constraints identified by Hu et al.—create a heterogeneous landscape rather than a uniform field. As a result, distinct regions within the plaque are subject to different selective pressures [49]. This structure is consistent with previous studies demonstrating the continuous generation, competition, and selection of genetic and phenotypic variants within spatially structured phage populations [32,51,52,53]. This interpretation is further supported by experimental observations demonstrating that phage populations can undergo rapid evolutionary adaptation in structured and in vivo environments, resulting in measurable shifts in infectivity and host interaction dynamics (e.g., Castledine et al., 2022 [32]). Consequently, phenotypic properties such as host range and killing efficacy should not be interpreted as fixed attributes of a single genotype. Instead, they should be interpreted as population-level distributions shaped by local ecological conditions and evolutionary dynamics. A plaque can therefore be viewed not as a static endpoint, but as an evolving ecological system in which outcomes are inherently probabilistic rather than deterministic [54,55]. This perspective provides a conceptual and biologically grounded framework for understanding phage population dynamics in the following sections.

To clarify the conceptual implications of the spatial structure introduced in this section, we summarize the key mechanisms in Box 1.

Box 1 **Conceptual expansion: The plaque as an evolutionary engine.**

This box expands mechanistically on the conceptual framework introduced in Section 2. Rather than a simple zone of killing, a plaque functions as a spatially structured evolutionary landscape driven by physical constraints [47,48].

(1) Physical Barriers Create Selective Gradients

As demonstrated by diffusion studies, phages cannot move freely within the plaque matrix. This physical limitation creates steep gradients, resulting in distinct ecological zones within a single plaque.

(2) Distinct Ecological Zones: Center vs. Periphery

The depletion of nutrients and host availability creates a structured environment where phage behaviors diverge. The contrasting dynamics are summarized in Table 1.

(3) Bacterial Refuges and Phenotypic Resistance

The spatial structure allows bacteria to survive in ways impossible in liquid culture. In low-MOI regions (the periphery), bacteria often rely on phenotypic resistance (such as transient dormancy or biofilm formation) rather than genetic mutation to evade attack [33,49]. These surviving pockets serve as reservoirs of bacterial diversity, forcing the phage population to continuously adapt [56].

(4) The “Snapshot” Fallacy

Standard lab assays often collapse this complex structure into a single “Yes/No” result. However, the observed phenotype reflects a probability distribution sampled from this heterogeneous population. Therefore, apparent inconsistencies in phage behavior are not errors but manifestations of the underlying population dynamics (see Section 3).

## 3. Host Range as a Probability Distribution

### 3.1. Resolving the “Snapshot Fallacy”

The structural complexity defined in Section 2 and Box 1 provides a conceptual basis for resolving a long-standing paradox in phage biology: the experimental discrepancy between plaque formation and liquid killing assays. The following discussion does not introduce a formally validated predictive model, but rather synthesizes existing experimental observations to provide an interpretative framework. It is a well-documented phenomenon that phages often exhibit bacterial suppression in liquid culture (spot tests) despite forming turbid or invisible plaques on the same host (Efficiency of Plating; EOP) [57]. Traditionally, these variations—often dismissed as “method-dependent artifacts”—have been treated as experimental noise. However, when viewed through a population-based lens, these observations can be interpreted not as contradictions but as consequences of the “Snapshot Fallacy” [57]. In this context, host range should not be considered a fixed binary property (Susceptible vs. Resistant), but rather a complex interaction shaped by the physical environment and population dynamics [58,59].

### 3.2. Visualizing the Shift

**Figure 3:**
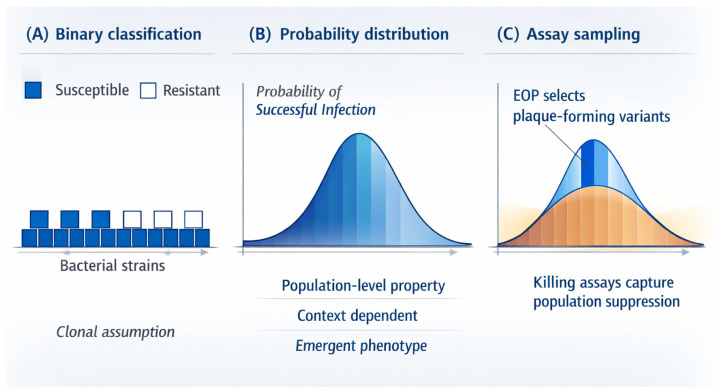
Host range as a probability distribution rather than a binary property. (**A**) Conventional representations classify bacterial strains as either susceptible or resistant to a given phage, implicitly assuming uniform infection outcomes based on binary logic. (**B**) In a population-based framework, host range emerges as a continuous probability distribution reflecting heterogeneous phage populations interacting with diverse bacterial states under variable conditions. (**C**) Different experimental assays sample distinct regions of the same underlying distribution: efficiency of plating preferentially captures high-fitness variants capable of macroscopic plaque formation, whereas killing assays reflect broader population-level suppression. Both measures therefore represent population-level outcomes rather than fixed properties of individual genotypes, and apparent discrepancies between assays arise from differential sampling rather than instability of phage properties. In panel C, the blue and orange shaded areas schematically indicate assay-dependent sampling regions within the underlying distribution and do not represent quantitative thresholds.

This shift from binary to continuous interpretation is illustrated in Figure 3.

- Figure 3A (The Clonal Assumption): Conventional representations impose sharp distinctions between “0” (resistant) and “1” (susceptible), implicitly assuming that all phage particles in a population behave identically.
- Figure 3B (The Probabilistic Reality): In contrast, host range can be interpreted as a probability distribution based on accumulated experimental observations [60]. Due to the population heterogeneity described in Section 2, a phage stock contains a spectrum of variants with varying infectivities. Infection outcomes are therefore inherently graded and context-dependent [45,58].

### 3.3. The Mechanism of Discrepancy: EOP vs. Killing

This conceptual framework provides a possible explanation for why different assays yield different results (Figure 3C). The key lies in the “physical constraints” discussed in Section 2 [59].

- Efficiency of Plating (EOP): Plaque formation is a stringent test. To form a macroscopic plaque, a phage variant must not only infect but also replicate rapidly enough to overcome the diffusion barriers of the agar matrix before the host reaches a stationary phase. Therefore, EOP can be interpreted as sampling the “high-fitness tail” of the distribution—the variants capable of overcoming physical constraints.
- Liquid Killing Assays: By contrast, liquid assays reduce these diffusion barriers. They integrate broader population-level effects, capturing “cooperative killing” and contributions from subdominant variants that may be too slow to form a visible plaque but are still effective in suppressing bacterial growth [52,57,61].

### 3.4. Implications for Reproducibility and Evolution

Interpreting host range as a probability distribution also provides a conceptual framework for understanding reproducibility and variability in phage experiments. Complete genetic uniformity is difficult to achieve in practice, and even populations derived from a single plaque may contain low-frequency variants. These variants, while often undetectable, may contribute to phenotypic variability under different environmental conditions. This inherent heterogeneity is consistent with the long-term co-evolution of phages and bacteria [40,62]. By maintaining a reservoir of diversity, phage populations may be better positioned to respond to environmental changes through standing genetic variation [50]. Therefore, therapeutic strategies that attempt to fix a single “optimal” genotype may, in some cases, limit adaptive potential. These considerations should be interpreted as a conceptual perspective derived from existing observations, and further experimental and computational validation will be required to assess their predictive value in therapeutic contexts.

## 4. Why Static Optimization Fails

Clinical and experimental evidence increasingly highlights the fragility of static phage formulations. In the pivotal PhagoBurn trial, a pre-configured cocktail showed decreased efficacy over time as bacterial populations in burn wounds rapidly evolved resistance [30]. Similarly, in vitro evolution studies consistently demonstrate that static phage preparations lose potency as host populations undergo phenotypic shifts, such as the formation of phage-resistant biofilms or metabolic dormancy [32,33]. Moreover, recent findings challenge the assumption that therapeutic phages should be strictly genetically uniform. Recent preprint work has suggested that phages may utilize “contingency loci” to generate localized hypervariability, representing a potential “bet-hedging” strategy to overcome host defenses (Gomez et al., 2025) [53]. These observations suggest that at least part of phage genetic diversity may arise from regulated mechanisms rather than purely stochastic variation, supporting the view that heterogeneity can contribute to functional adaptability. Despite this, conventional approaches continue to assume that therapeutic efficacy can be optimized by fixing a single, well-defined genotype.

However, as demonstrated in Section 2 and Section 3, key phage properties—including host range and killing efficacy—do not arise from fixed attributes of individual genotypes. Instead, they emerge from population-level dynamics operating within structured and evolving systems [55,63]. Under these conditions, static optimization tends to capture only a narrow region of a broader and continuously shifting phenotypic distribution (Figure 3). Importantly, the failure of static optimization does not reflect excessive variability or insufficient manufacturing control. Rather, it reflects a conceptual error: treating a dynamic, adaptive biological system as if it were a static object [39]. Attempts to eliminate variability entirely may risk removing the very diversity that enables phage populations to maintain functionality across heterogeneous and changing host environments [39,40]. From this perspective, reproducibility cannot be defined by strict genotypic uniformity. Instead, reproducibility must be understood at the level of population behavior—specifically, the maintenance of stable distributions of efficacy within defined boundaries [54]. To achieve this, a transition is required from selecting “optimal” clones to designing evolutionarily robust populations that can navigate the host’s adaptive landscape [54,64].

## 5. Regulatory Constraints and Structural Mismatch

### 5.1. The CMC Dilemma: Binary Regulations vs. Biological Probabilities

The fundamental friction in phage therapy regulation originates at the level of Chemistry, Manufacturing, and Controls (CMC). Traditional pharmacopeial standards rely on the principle of “sameness,” where a therapeutic product must demonstrate strictly defined identity to a reference standard across batches [36,38,65]. However, enforcing absolute genomic invariance may conflict with the biological reality of viral populations. High-throughput sequencing analyses have revealed that even within a “purified” phage stock, quasi-species diversity exists and can fluctuate during manufacturing [61]. More critically, this regulatory insistence on binary definitions—treating a phage as simply “present” or “absent,” and a bacterium as “susceptible” or “resistant”—fails to capture the true nature of phage-host interactions. As illustrated in Section 4 (Figure 3), host range is not a binary switch but a continuous probability distribution [59]. Under current strict Good Manufacturing Practice (GMP), the natural fluctuations of this distribution are often categorized as “impurities” or manufacturing deviations. This creates a “Quality Paradox”: processes designed to ensure safety may inadvertently filter out the biological adaptability required for efficacy [61,65].

### 5.2. Bottom-Up Pressures: The Magistral and Compassionate Use Models

While centralized regulatory agencies debate theoretical frameworks, clinical practice has developed pragmatic, bottom-up solutions. The “Magistral Phage” framework in Belgium represents the most successful operational model, treating phages not as industrially manufactured goods but as personalized medicinal formulas compounded by pharmacists [37,38,66]. This approach bypasses the rigid requirements of centralized marketing authorization by shifting responsibility to the compounding professional. Similarly, in the United States and Australia, the proliferation of expanded access (compassionate use) has generated a significant volume of Real-World Evidence (RWE). Dedrick et al. (2023) demonstrated that safe and effective treatment is possible for drug-resistant *Mycobacterium* infections using personalized cocktails, without traditional fixed-product standardization [34]. Furthermore, the STAMP protocol in Australia has standardized the *process* of treatment monitoring rather than the *product* itself [35]. Crucially, the data generated from these localized treatments challenge the “top-down” insistence on broad-spectrum, fixed cocktails [37,38,42].

### 5.3. Institutional Evolution: Moving to Implementation (2024–2025)

**Figure 4:**
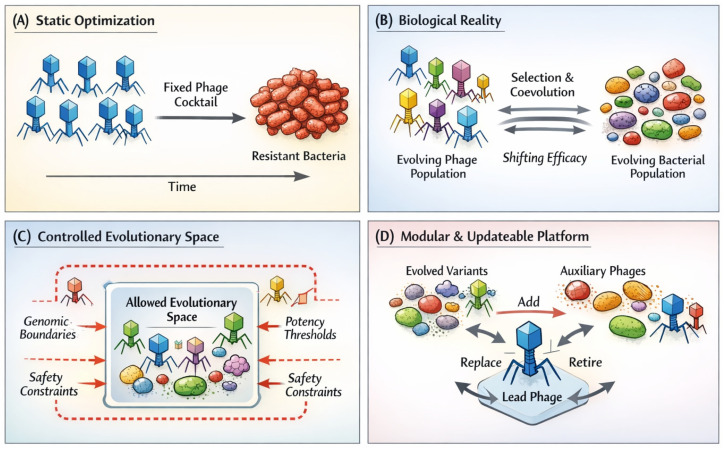
Controlled evolutionary platforms reconcile phage biology with therapeutic and regulatory requirements. (**A**) Conventional phage development relies on static optimization, in which a single phage genotype or a fixed cocktail composition is selected and maintained over time. Although initially effective, such static formulations lack evolutionary robustness under selective pressure as bacterial populations adapt. (**B**) In biological reality, both bacterial and phage populations evolve dynamically. Therapeutic efficacy therefore emerges as a shifting population-level distribution rather than a fixed property of individual genotypes. (**C**) Controlled evolutionary platforms harness adaptation within predefined boundaries. Evolutionary change is biologically unavoidable but need not be unbounded. Regulatory and quality control efforts focus on ensuring that population-level behavior remains confined within acceptable functional and safety limits, rather than suppressing individual mutations. (**D**) Within this framework, phage therapy is implemented as a modular and updatable system. Stable lead phages provide a consistent safety core, while evolved variants or auxiliary phages can be added, replaced, or retired according to predefined criteria. Product identity is thus defined at the population level, enabling governed adaptability while preserving safety, predictability, and long-term efficacy. This framework emphasizes control over evolutionary processes rather than suppression of evolutionary change.

In response to these bottom-up pressures and the accumulation of clinical data, major regulatory bodies are moving from “discussion” to “implementation.” The focus is shifting away from the “Static Optimization” model shown in Figure 4A, which assumes a fixed product can indefinitely treat a changing target.

- United Kingdom (2024): A landmark report by the House of Commons Science, Innovation and Technology Committee explicitly recommended establishing a dedicated regulatory pathway for phages, recognizing that they cannot be regulated simply as “biological chemicals” [67].
- Portugal (2024): The Portuguese regulatory authority (INFARMED) approved Deliberação 112/CD/2024, enabling the hospital use of individualized magistral bacteriophage preparations. This framework reflects principles similar to the Belgian magistral framework, as also discussed in recent analyses of health system integration, suggesting that structured approaches to personalized phage therapy can be implemented across different regulatory environments [68].
- Germany (PhagoFlow Project, 2019–2024): The publicly funded PhagoFlow initiative provides a representative example of efforts to operationalize individualized phage therapy within a clinical framework. The project investigates the feasibility of producing patient-specific phage preparations within hospital pharmacies in clinically relevant timeframes, integrating purified phage active pharmaceutical ingredients (APIs), phagogram-guided selection, and on-site compounding. While not designed as a conventional clinical trial, it establishes a practical workflow for the personalized adaptation of phage preparations, particularly for multidrug-resistant infections such as *Pseudomonas aeruginosa*. Importantly, it illustrates how individualized phage therapy can be implemented under individual treatment trial frameworks within existing healthcare infrastructures [69].
- United States (2024): The FDA is operationalizing the “Platform Technology” provisions from the Food and Drug Omnibus Reform Act (FDORA). This allows phage libraries to be treated as adaptable platforms, permitting updates to the viral strains without restarting the entire approval process [70].
- Japan (2025): The Pharmaceuticals and Medical Devices Agency (PMDA) has taken a leading role by publishing “Regulatory considerations for developing phage therapy medicinal products.” Based on expert consultations conducted throughout 2024–2025, this document clarifies quality control and safety assessment standards, marking Japan’s transition from observation to active regulatory definition [71].

### 5.4. Synthesis: Governing Process over Product

The trajectory of phage regulation is shifting from an “identity-based” paradigm to a “process-based” paradigm. The structural mismatch identified in earlier critiques—represented by the gap between Figure 4A,B—is being resolved not by forcing phages to behave like chemicals, but by adapting regulations to govern biological dynamics. The emerging consensus suggests that future approval pathways will focus on validating the system—the quality of the library, the accuracy of the companion diagnostic, and the safety of the production process—rather than the momentary genetic sequence of a specific phage [36]. In this framework, evolutionary variability is no longer a defect to be suppressed, but a parameter to be monitored and controlled [64]. This shift towards “evolutionary pharmacology” ensures that reproducibility is defined by stable clinical outcomes rather than static genomic sequences [54].

## 6. Toward Controlled Evolutionary Platforms

The regulatory shifts described in Section 5 lead to a critical insight: the question is no longer whether phage therapy can be regulated, but how regulatory systems can evolve to accommodate biological adaptation. This realization motivates a new design framework in which phage products can be understood not as static entities, but as controlled evolutionary platforms (Figure 4) [66,70].

We acknowledge that real-world environments, including clinical and agricultural settings, introduce additional layers of complexity that are not fully captured in simplified laboratory models. Factors such as host immune responses, microbiome interactions, spatial heterogeneity, and treatment conditions can influence therapeutic outcomes in ways that are difficult to predict from controlled experiments alone. Accordingly, the framework presented here should be interpreted as a conceptual and guiding model rather than a fully predictive system. Future implementation will require integration of multi-layered datasets, including phage genomes, bacterial host genomes, and metagenomic context, to enable more realistic and adaptive modeling. Importantly, elements of this approach are already supported by experimental and clinical observations demonstrating that phage-driven selection can influence bacterial phenotypes and evolutionary trajectories in therapeutically relevant ways [34,35,64]. At the core of this platform is a clear distinction between the mere presence of variability and the governance of variability. Evolutionary change in phage populations is biologically unavoidable; attempting to halt it completely is unlikely to succeed and may even undermine therapeutic robustness [39,72]. However, inevitability does not imply disorder. Safety and efficacy do not require the elimination of evolution, but rather the definition of acceptable boundaries within which adaptive change is permitted to occur [54,73]. Crucially, such governance does not imply that evolution is left to proceed as an entirely unguided process (Figure 4B). Accumulating experimental and theoretical work suggests that coevolutionary dynamics can, in practice, be intentionally shaped so that bacterial adaptation proceeds along constrained and clinically favorable trajectories. In this context, evolutionary responses to phage pressure can be redirected toward paths in which resistance acquisition is tightly coupled to substantial fitness costs, including restored antibiotic susceptibility or attenuated virulence [74,75]. Recent studies have begun to operationalize this principle by actively intervening in spontaneous evolutionary selection, thereby steering host–phage coevolution toward defined therapeutic gains rather than merely observing resistance emergence [64,76,77]. From this perspective, adaptive change within the phage–bacteria system is not a failure mode to be suppressed, but a controllable variable that can be harnessed to maintain long-term therapeutic performance. Within this framework, regulatory control shifts from suppressing individual mutations to enforcing a two-tiered boundary system (see Box 2): Negative boundaries define strict prohibitions on unsafe traits, such as the acquisition of toxin genes, lysogeny-associated functions, or antimicrobial resistance determinants. In contrast, permissive boundaries delineate genomic and functional regions in which adaptive variation is expected and allowed to occur. Accordingly, product identity is no longer defined by a single immutable genotype, but by a constrained distribution of variants operating within predefined safety and performance limits (Figure 4C). This population-level definition of identity allows controlled adaptability while preserving predictability and regulatory oversight. A second defining feature of controlled evolutionary platforms is modularity. As illustrated in Figure 4D, phage therapeutics can be structured around stable lead phages that provide a consistent safety profile and functional backbone, while allowing auxiliary modules—such as receptor-binding proteins or tail fiber components—to evolve or be exchanged in response to bacterial surface variation [62]. Importantly, adaptive updates occurring within permissive boundaries are not conceptualized as new drugs requiring de novo authorization. Instead, they are managed through simplified notification-based pathways, supported by genomic quality control and functional verification. This framework is not purely theoretical. Elements of it are already implemented in directed evolution protocols and adaptive cocktail strategies used in both experimental and clinical contexts [34,35]. What has been lacking is an integrated regulatory logic that formally recognizes and governs such practices. By shifting regulatory emphasis from static identity to controlled process, controlled evolutionary platforms provide that missing logic. Finally, this approach reframes the economic model of phage therapy. At first glance, continuous monitoring and adaptive updating may appear to increase operational complexity. However, this view overlooks the hidden cost of static formulations: rapid therapeutic obsolescence driven by bacterial evolution [42]. Static phage products inevitably lose efficacy, necessitating repeated redevelopment. In contrast, controlled evolutionary platforms internalize adaptation as a routine and regulated process, thereby preserving long-term therapeutic relevance. In doing so, phage therapy is transformed from a disposable intervention into a sustainable, renewable therapeutic infrastructure [64].

Despite the conceptual strengths of this framework, several limitations and uncertainties should be acknowledged. First, evolutionary trajectories in complex biological systems are inherently difficult to predict, particularly in clinical and environmental settings where multiple selective pressures interact simultaneously. Second, while population-level control offers a promising regulatory perspective, practical implementation will require robust standardization of monitoring strategies and data integration. Third, not all infections may benefit from adaptive phage strategies, particularly in acute settings where rapid and predictable bacterial clearance is required. At the same time, it should be noted that therapeutic outcomes may inherently emerge from the interplay between phage evolution, bacterial adaptation, and host immune responses, as suggested by observations from successful phage therapy cases. These considerations highlight that the controlled evolutionary platform should be viewed not as a universal solution, but as a context-dependent strategy that complements, rather than replaces, existing antimicrobial approaches.

To translate the conceptual framework of controlled evolutionary platforms into practical design principles, we summarize the key elements in Box 2.

Box 2 **Design framework: controlled evolutionary phage therapy.**

To make the “Controlled Evolutionary Platform” concept work in practice [70], we propose a set of clear boundary definitions. These distinguish between the fixed safety foundation and the flexible parts allowed to adapt.

(1) The Safety Core (Negative Boundaries: What Must Not Change)

The “Lead Phage” (the platform’s foundation) must satisfy strict, immutable safety rules. Regardless of any evolutionary updates, the phage population is strictly prohibited from acquiring:

- Harmful Genes: Absolute absence of genes that integrate into the bacterial genome (lysogeny-related genes like integrases) or genes encoding toxins and antibiotic resistance [61,78].
- Functional Failures: The core scaffold must maintain its baseline ability to replicate and evade common bacterial defenses (e.g., CRISPR-Cas), ensuring the platform remains viable even as it updates its targeting mechanism [54,63].

(2) The Adaptive Periphery (Permissive Boundaries: What Is Allowed to Evolve) Evolutionary variation is explicitly permitted, but only within specific “safe zones” of the genome:

- Targeted Variability: Mutations are expected and allowed within the Receptor Binding Proteins (RBPs/Tail fibers). This allows the phage to track shifting bacterial surface antigens, similar to how flu vaccines are updated [62].
- Backbone Stability: Outside of these specific targeting regions, the rest of the genome must remain highly stable (e.g., >95–99% identity to the reference strain) to ensure predictable pharmacokinetics [79,80].
- Phenotypic Consistency: The updated variants must act like the parent strain—killing the bacteria efficiently without becoming toxic to human cells [61].

(3) Regulatory Simplification (The Notification Pathway)

Under this framework, variants that stay within the boundaries above are treated as “lineage updates,” not new drugs.

- No New Full Review: Because the “Safety Core” remains unchanged, updates driven by RBP mutations do not require a full toxicological or clinical re-evaluation [36].
- Fast-Track Release: Release is authorized based on whole genome sequencing (WGS). This confirms that changes are confined to the “Adaptive Periphery” and that no “Negative Boundaries” have been breached.
- Sustainability: This simplified mechanism reduces the cost and time of updates, making it economically feasible to keep the phage therapy effective over the long term [42].

## 7. Materials and Methods

Generative AI, specifically ChatGPT (GPT-5.3; accessed on April 2026), was used for figure conceptualization and language refinement. All scientific content and interpretations were verified by the authors.

## 8. Conclusions: Phage Therapy in Schrödinger’s Box

Phage therapy stands at a unique crossroads in modern medicine. It is neither a static chemical drug nor an unconstrained biological accident. Rather, it exists at the intersection of design and evolution. Throughout this review, we have argued that the challenges of phage therapy largely arise from a fundamental conceptual barrier—an evaluation mismatch between biological reality and conventional frameworks [36,37,38,42].

The traditional pharmaceutical model forces us to treat a phenomenon that is inherently a dynamic “movie” as if it were a static “snapshot.” This is where the metaphor of Schrödinger’s box is most poignant. When we confine phages within rigid assumptions of “fixed identity,” they appear unstable and problematic. However, once we “open the box” and accept phages as evolving populations, the paradox dissolves.

To move forward, we must redefine the “consistency” we seek. It resides not in the strict maintenance of a fixed nucleotide sequence, but in the reliable performance of population dynamics. By adopting the Controlled Evolutionary Platform framework (Section 6), we can operationalize this concept. Distinguishing between the “Safety Core” (which anchors safety) and the “Adaptive Periphery” (which tracks bacterial evolution) allows regulators to shift from a paradigm of “prohibiting change” to “managing updates” [66,70].

This shift aligns therapeutic strategy with deep biological history: biological systems persist not by remaining static, but by continuously adapting to changing environments [81]. For over three billion years, bacteria and phages have co-survived through such dynamic interactions. In contrast, the antibiotic era attempted to stop this dynamic engine with static walls—and those walls are now crumbling [82]. We have been stuck in an exhausting game of “catch-up,” manually developing new drugs to replace those that fail [83,84]. To break this cycle, we must harness the sustainable co-evolutionary dynamics of nature [42]. Opening Schrödinger’s box does not reveal chaos; it reveals the opportunity to align biological reality with therapeutic design. Embracing this perspective—moving from static snapshots to dynamic consistency—will allow phage therapy to mature from a niche alternative into a sustainable, scalable, and evolutionarily robust pillar of modern medicine [54,66,74].

To clarify the intended interpretation and limitations of Schrödinger’s cat metaphor used in this review, we provide a conceptual note in Box 3.

Box 3 **Conceptual note: interpreting Schrödinger’s metaphor in phage therapy.**

The Metaphor: In this review, we employ the metaphor of Schrödinger’s cat strictly as a conceptual analogy. Why use this metaphor? It illustrates that a phage population is not a single, fixed object like a small chemical molecule. Instead, it encompasses a range of functional states simultaneously. When we measure a phage’s host range or killing activity, we are not measuring a binary property (on/off); we are sampling a specific outcome from an underlying probability distribution (see Figure 3) [57,59,60].

Order, not Chaos: Crucially, this analogy does not imply that phages are unpredictable. The “distribution” of phage functions is shaped by strict biological constraints and evolutionary trade-offs [46,72]. Therefore, variability is bounded and interpretable.

The Conceptual Barrier: The challenge for clinical application lies not in a lack of phage efficacy, but in an “evaluation mismatch.” The traditional “fixed pharmaceutical” model forces us to treat a phenomenon that is inherently a dynamic “movie” as if it were a static “snapshot.” This conceptual barrier is the root cause of the apparent unpredictability in clinical results and arguably hinders the full exploitation of the phage’s inherent adaptive capabilities. To move forward, we must fundamentally redefine the “consistency” we seek: it resides not in a fixed nucleotide sequence, but in the reliable performance of population dynamics.

## Figures and Tables

**Table 1:** Ecological and evolutionary contrasts between plaque center and periphery.

**Feature**	**Plaque Center (Resource-Depleted)**	**Plaque Periphery** **(Active Front)**
**Nutrient State**	Depleted: Resources are exhausted due to prior bacterial growth and lysis.	Abundant: Nutrients are available, supporting active bacterial metabolism.
**Host Physiology**	Dormant or Lysed: Hosts are either dead or in a stationary/starved state.	Exponential Growth: Hosts are metabolically active and dividing.
**Phage Dynamics**	High Density/Stagnation: Phages are crowded but trapped. High competition for survival.	Low Density/Amplification: Low MOI interactions drive rapid rounds of infection.
**Selection Pressure**	Selection for stability, persistence, and decay resistance.	Selection for rapid adsorption, shorter latent periods, large burst sizes, and altered host-interaction dynamics [33,43,44,46].

## Data Availability

No new data were created or analyzed in this study. Data sharing is not applicable to this article.
